# If Not Now, When? the Role of Geriatric Leadership as Covid-19 Brings the World to Its Knees

**DOI:** 10.3389/fmed.2020.00232

**Published:** 2020-05-15

**Authors:** Tzvi Dwolatzky

**Affiliations:** ^1^Geriatric Unit, Rambam Health Care Campus, Haifa, Israel; ^2^Ruth and Bruce Rappaport Faculty of Medicine, Technion, Israel Institute of Technology, Haifa, Israel

**Keywords:** COVID-19, pandemic, geriatrics, aging, health care

## Introduction

The Covid-19 virus is a ruthless enemy that knows no borders. The measures that governments have been forced to take are devastating economic life and exposing major inadequacies in health care systems. While the global village dissipates with the halting of international travel, people are facing lockdown in their homes in a desperate effort to curtail the spread of this virulent virus. Not surprisingly, those who are older, sicker, frail, and socially isolated, are bearing the brunt of this attack. The respiratory sequelae of Covid-19 in the older population are severe, and many require mechanical ventilatory support in intensive care units. Mortality is high, and for those who survive recovery is slow. The need to treat a vast number of patients is overwhelming, resources are scarce, and difficult ethical decisions have to be made. Triage criteria are being developed and are generally designed out of urgent necessity rather than being based on clear evidence-based scientific criteria (1).

As the world struggles to cope with the worst viral pandemic of the last century, a recent report from Spain shocked the reader with a new reality.

## Coronavirus: Elderly Found ‘Dead and Abandoned’ in Spanish Nursing Homes

*Elderly people have been found dead and abandoned in nursing homes in Spain, the country's defence minister has said. Margarita Robles, speaking in a television interview, said the army had made the discoveries while disinfecting old people's homes. The military had found “the elderly absolutely abandoned, if not dead in their beds”, said Robles* (2).

What went wrong? How did a tragedy like this happen in a country that was rated by Bloomberg as the world's healthiest nation in 2019? (3).

We certainly are not here to judge, and this specific incident is under investigation by the authorities. However, one may postulate regarding the reasons for such a tragic situation. One must remember that this event occurred in a very unusual situation, where the rapid spread of disease has ruthlessly destroyed infrastructure as health care needs outstrip resources. One may try to understand the personal perspective of health care workers pushed to their physical and mental limits in providing care to old and frail people at a time of crisis, while harboring their own concerns and fears. Indeed, providing care for patients in an environment where the rapid spread of a highly infectious disease certainly places the health care worker at significant personal risk. Moreover, in an atmosphere where the treatment of older functionally or cognitively impaired older people is considered to be futile, the door to abandonment is wide open.

However, this event must serve as a wake-up call for us all—individuals, families, health care workers, policy makers, governments. As I was taught as a resident undergoing a compulsory Advanced Cardiac Life Support Course, the first thing that one should do when faced with a resuscitation is to “take your own pulse.” As health care workers, our first responsibility is to ensure that we are personally prepared both physically and mentally to go out to war against a deadly virus. On answering the call to go out to battle, our role is to save lives where possible, yet always to maintain human dignity and respect and alleviate suffering.

This call lies at the very heart of Geriatric Medicine. In the mid-twentieth century, Marjory Warren, who is regarded as the pioneer of Geriatric Medicine, and was co-founder of the Medical Society for the Care of the Elderly (later becoming the British Geriatrics Society), fought for the medical recognition of the neglected older population. She recognized that older people had different needs, and emphasized a multidisciplinary comprehensive rehabilitative approach that forms the basis of the profession today (4). Clearly, Warren was a visionary, a pioneer and a leader. Generations of prominent geriatricians have followed, and Geriatric Medicine is a recognized medical specialty in most countries. And now, with a viral pandemic sweeping across the globe, geriatricians are actively involved in the clinical care of vast numbers of older people in the community and in hospital settings. Yet, geriatricians must take on another role in the fight against coronavirus, a role of leadership.

## The Geriatrician as a Leader in the COVID-19 Pandemic

*For if you remain silent at this time, relief and deliverance …. will arise from another place, but you and your father's family will perish. And who knows but that you have come to your royal position for such a time as this?”* (5).

It is at a time like this that geriatricians must step in to take the lead. It is imperative that we identify issues affecting the health and well-being of older people, actively promote awareness, and work to influence policy at both local and national levels. I will relate to some of the central issues that should be addressed.

### The Effect of Lockdown on Older People

At the time of writing, a third of the global population is on coronavirus lockdown (6). Social distancing and the restriction of movement, with a clear call to stay at home and thus prevent exposure to other people who may be a source of coronavirus infection, is in accordance with the World Health Organization's efforts to limit the spread of the virus. However, lockdown has major repercussions on the lives of older people.

### Health Maintenance

For older people who frequently suffer from a number of chronic conditions, health maintenance is essential. Adequate control of factors such as blood glucose, blood pressure, cardiac failure, mobility in Parkinson's disease, chronic pain, and many others, is essential in promoting well-being and preventing complications. With the initiation of lockdown, older people are unable to visit their family physician for checkups or to receive prescriptions, and they have limited ability to get to the pharmacy or access other medical services such as physical therapy. As the time spent in lockdown progresses, the likelihood is that many older people will develop unnecessary complications due to poor control of chronic illnesses.

To prevent this a “reach-out” policy should be developed, based on the traditional and effective standardized multidimensional comprehensive geriatric assessment. Such an approach will help to minimize the development of harmful geriatric syndromes, such as falls, frailty and polypharmacy. Community clinics should contact older patients regularly to enquire about health status, and should obtain information regarding measurable physical signs, adequate supplies of medications, and other health needs. Technology can play an important role in health monitoring by the use of smart phone applications, telemedicine, and other modalities. In addition, older people who are living alone should be encouraged to install fall detection devices, and to use wearable “call for help” pendants or wristwatches.

### Psychosocial Isolation

The obvious result of lockdown is psychosocial isolation. Most younger people or those with families manage to adapt to the stresses of social isolation. But for older people who are often alone and functionally limited, the lack of social contact can be devastating. Contact with family members and friends is discouraged as part of the call for social distancing. The effect of loneliness on one's mental state at an older age has been clearly determined. The path to depression, anxiety and cognitive decline is often inevitable. A lack of appetite, limited food supplies, and reduced motivation to prepare adequate meals, will likely result in a deterioration of the older person's nutritional status.

In an effort to alleviate these untoward results, local agencies in cooperation with volunteer organizations should identify older people who are alone at home. These people must be contacted regularly to “touch base” regarding their needs and to help them replenish dwindling supplies. They should be offered “meals on wheels” and home delivery of provisions. Regular telephone contact, discussions with neighbors at a distance “over the balcony rails,” and videoconferencing with family and friends is encouraged. It is essential to maintain mental function by reading, solving crossword puzzles or sudoku, and engaging in other cognitively stimulating activities such as scrabble, chess or bridge, especially by partnering with other people on-line. Older people should be encouraged to adopt a daily exercise schedule to include personal preferred forms of activity, such as stretching and isometric exercises, and walks around the house a number of times during the course of the day.

### Health Priority of Older People

Current experience with the Covid-19 outbreak clearly indicates that older people are at a markedly increased risk for complications and mortality. It has been shown that mortality begins to increase from the age of 60 years, rapidly rising to 21.9% in confirmed cases above the age of 80 (7). The respiratory manifestations of this disease are severe and frequently require mechanical ventilation in high care and intensive care units. As such, every measure that will decrease the exposure of older people to coronavirus should be adopted, and an approach of early detection and treatment should be adopted.

### Minimizing Exposure to Coronavirus for Older People and Health Workers

Older people who are cognitively and/or functionally impaired and are living at home are usually cared for by nursing assistants. The continuing employment of health workers is essential at the time of crisis. In Italy, healthcare workers constitute 9% of Covid-19 patients. Thus, protecting these workers must be a main priority. As such all health workers should be given appropriate training and protective equipment to prevent infection. In addition, this sector must be given priority for Covid-19 testing. Not only will this ensure the rights and personal safety of the workers, but it will limit the exposure of the older population to the virus.

### Preventing Tragedies in Nursing Homes

As the Covid-19 pandemic unfolds, the tragedy of a rapid spread of the virus among frail and vulnerable older residents of nursing homes has resulted in catastrophic consequences. The Centers for Disease Control and Prevention (CDC) has issued clear guidelines for protecting residents, families and staff of long-term facilities from serious illness, complications, and death (8). The strategies include closing off the facility by restricting visitors, the use of personal protective equipment, the active screening of residents and staff, the implementation of social distancing and isolation of suspected cases, and the early identification, and treatment of severe illness. Geriatricians and gerontologists should spearhead the implementation of these key strategies in nursing homes.

### Therapeutic Priority

As yet there is no proven vaccine or therapeutic agent for Covid-19. However, a number of agents are being used empirically based on clinical experience, albeit with limited supportive evidence. These include hydroxychloroquine sulfate and zinc. There is also some interest in using the interleukin-6 inhibitor tocilizumab, which has recently been approved by the US Food and Drug Administration (FDA) in a phase 3 trial for severely ill Covid-19 patients hospitalized with pneumonia (9). Based on the knowledge that the older population is at the greatest risk, priority should be given for providing therapeutic agents particularly to older people with coronavirus infection, as much as this is possible considering local policy and availability.

### Ageism and the Rights of Older People

The unfolding coronavirus pandemic has pushed health systems way beyond their limits. Demand has rapidly surpassed supplies in many countries. This has resulted in a chaotic situation where difficult decisions have to be made. Probably the most painful of these decisions is who should be entitled to the use of mechanical ventilators. Policy makers have rapidly designed triage systems in order to provide scarce life-saving equipment to those most likely to benefit. Considering that the prevalence of major complications is significantly higher in older people in whom the chances of survival are lower, restrictions have been developed based specifically on chronological age.

Geriatricians should raise their voices in opposition to such a manifestation of ageism. For years chronological age was used as an absolute criterion for withholding critical and life-saving services from older people. Treatment in Intensive Care Units, the provision of hemodialysis, and surgical interventions, as a few examples, were not provided to those over the age of 70 due to limited availability. Decades of research, education, and lobbying by geriatricians, have convinced the medical world that one should relate to the physiological and functional state of the older person as a measure of biological age rather than to the absolute criterion of chronological age. When faced with difficult decisions due to a lack of resources we must consider age in the context of comorbidities and function.

### Autonomy and Personal Medical Preferences

Autonomy is one of the four pillars of bioethics. People have the right to determine their own destiny, and this right must be respected. In the throes of a spreading pandemic, there is a greater likelihood that an older person will have to face difficult decisions regarding life-saving measures. As such geriatricians should encourage patients to express their medical preferences in a living will.

### A Final Reflection

As geriatricians we are proud of our role as leaders of the medical teams caring for our older members of society. While countries fight for survival in the battle against coronavirus, we must lead the effort to ensure that older people are not forgotten, that their needs are provided, and that they are treated with the respect that they deserve. And if not now, when?

*Hillel said: … If not now, when?* (10).

## Author Contributions

TD developed the concept of the article and wrote the manuscript.

## Conflict of Interest

The author declares that the research was conducted in the absence of any commercial or financial relationships that could be construed as a potential conflict of interest.
